# The Development of Public Policies to Address Non-communicable Diseases in the Caribbean Country of Barbados: The Importance of Problem Framing and Policy Entrepreneurs

**DOI:** 10.15171/ijhpm.2016.74

**Published:** 2016-06-15

**Authors:** Nigel Unwin, T. Alafia Samuels, Trevor Hassell, Ross C. Brownson, Cornelia Guell

**Affiliations:** ^1^Chronic Disease Research Centre, University of the West Indies, Bridgetown, Barbados.; ^2^MRC Epidemiology Unit and UKCRC Centre for Diet and Activity Research, University of Cambridge, Cambridge, UK.; ^3^Healthy Caribbean Coalition, Bridgetown, Barbados.; ^4^Prevention Research Center in St. Louis, Brown School, Washington University in St. Louis, St. Louis, MO, USA.; ^5^Division of Public Health Sciences and Alvin J. Siteman Cancer Center, Department of Surgery, Washington University School of Medicine, Washington University in St. Louis, St. Louis, MO, USA.

**Keywords:** Public Policy, Non-Communicable Diseases (NCDs), Multiple Streams, Policy Entrepreneurs

## Abstract

**Background:** Government policy measures have a key role to play in the prevention and control of non-communicable diseases (NCDs). The Caribbean, a middle-income region, has the highest per capita burden of NCDs in the Americas. Our aim was to examine policy development and implementation between the years 2000 and 2013 on NCD prevention and control in Barbados, and to investigate factors promoting, and hindering, success.

**Methods:** A qualitative case study design was used involving a structured policy document review and semi-structured interviews with key informants, identified through stakeholder analysis and ‘cascading.’ Documents were abstracted into a standard form. Interviews were recorded, transcribed verbatim and underwent framework analysis, guided by the multiple streams framework (MSF). There were 25 key informants, from the Ministry of Health (MoH), other government Ministries, civil society organisations, and the private sector.

**Results:** A significant policy window opened between 2005 and 2007 in which new posts to address NCDs were created in the MoH, and a government supported multi-sectoral national NCD commission was established. Factors contributing to this government commitment and funding included a high level of awareness, throughout society, of the NCD burden, including media coverage of local research findings; the availability of policy recommendations by international bodies that could be adopted locally, notably the framework convention on tobacco control (FCTC); and the activities of local highly respected policy entrepreneurs with access to senior politicians, who were able to bring together political concern for the problem with potential policy solutions. However, factors were also identified that hindered multi-sectoral policy development in several areas, including around nutrition, physical activity, and alcohol. These included a lack of consensus (valence) on the nature of the problem, often framed as being predominantly one of individuals needing to take responsibility for their health rather than requiring government-led environmental changes; lack of appropriate detailed policy guidance for local adaptation; conflicts with other political priorities, such as production and export of alcohol, and political reluctance to use legislative and fiscal measures.

**Conclusion:** The study’s findings indicate mechanisms to promote and support NCD policy development in the Caribbean and similar settings.

## Background

Globally, chronic non-communicable diseases (NCDs) account for over 60% of deaths, and over two-thirds of these occur in low- and middle-income countries (LMICs).^1^ In the countries of the Americas, with one exception (Haiti), NCDs account for three quarters or more of all deaths.^2^ The Caribbean experiences a particularly high burden of NCDs. Age standardised mortality rates from NCDs in English speaking Caribbean countries range from thirty percent higher to over twice as high as those in North America.^3^ Underlying this high mortality are high levels of risk factors, particularly obesity, diabetes, and hypertension.^4^

Effectively preventing and managing NCDs requires appropriately designed health systems and responses from many sectors outside health, including trade, finance, agriculture, education, urban planning, and education.^5^ Public policy has a key role to play, and this is reflected in the policy recommendations in the Global Action Plan on NCDs,^5^ which arose out of the 2011 United Nations (UN) high level meeting.^6^ Four years before the UN meeting, Heads of Government of the Caribbean Community (known as CARICOM) held a regional summit on NCDs, the first of its type in the world, and notably in a middle-income region. The outcome of this meeting was the 2007 Port of Spain Declaration on NCDs,^7^ in which governments committed to a range of multi-sectoral policy measures for NCD prevention and control.

Given the recognised importance of policy measures to reduce the burden of NCDs, it is perhaps surprising that there is relatively little research, particularly from low- and middle-income regions, addressing their implementation and effectiveness. The process of policy-making and implementation is complex, rarely if ever being based on simple rational choice and following the textbook ‘stages heuristic’ policy cycle.^8,9^ In addition, the systems within which NCD risk factors, such as obesity, arise are also complex,^10^ meaning that even well-implemented policy measures may not always have the effect that was intended or found in other settings. In recognition of these complexities, the question of what works in terms of policy is now often posed as, ‘what works for whom, in what contexts and through what mechanisms?’^11,12^

The aim of this article is to contribute to an overall goal of understanding the successes and difficulties of formulating and implementing policy on NCDs in the Caribbean and similar developing regions. The work was undertaken in Barbados, a member of CARICOM, known to have made a major contribution to the 2007 Port of Spain Declaration on NCDs and perceived within the region as being highly active in NCD prevention and control activities. Barbados is an independent nation with a resident population of 286 000. It is a developing economy,^13^ a member of the UN conference of small island developing states,^13^ and has an estimated per capita gross national income of US$14 880.^14^ Barbados is a constitutional democracy, with elections held at least every 5 years for its 30 member House of Assembly. The last election was in 2013, and prior to that, 2008. Citizens of Barbados have access to government healthcare facilities that are free or highly subsidised at the point of use. There is also a highly active private primary healthcare sector. Over 80% of all deaths are due to NCDs, with around 40% of these occurring before the age of 70.^15^ Forty percent of the adult (>25 years) population is hypertensive, 19% has diabetes, and two-thirds is overweight or obese.^16^ As a member of CARICOM, Barbados is a signatory of the 2007 regional declaration on NCDs^7^ and has been closely involved in the World Health Organization’s (WHO’s) recent responses to NCDs; for example, Barbados’ Chief Medical Officer chaired the WHO Executive Board for two years until May 2013.^17^

The objectives of the work were to describe for the period 2000 and 2013 the content of stated policy, the extent to which policy statements are perceived to have been implemented and to investigate what has worked well and why, and what has not worked well and why. We chose the year 2000 onwards as our frame of reference to examine policy developments as this timeframe fitted well with perceived key national and regional policy initiatives addressing NCDs.

## Methods

### Design and Theoretical Framework

We used a qualitative case study design involving a structured document review of relevant policy documents and semi-structured interviews with key informants from government, civil society, and the private sector. This design allowed us to compare stated policies, agenda-setting and planning in key documents with informants’ narratives on the process of formulating government-led NCD policies, and their knowledge, insight and experiences on the current state of implementation of policies. The subject matter of this study is government-led policy, what can also be called ‘public policy,’^18^ for NCDs. A classic definition of public policy is ‘anything a government chooses to do or not to do,’^19^ referring to all levels of government.^20^ The definition draws attention to the fact that ‘policy’ is much more than what is contained in ‘policy documents’ or legislation. The pragmatic definition of NCD public policies for the work reported here is also broad. It is adapted from Buse and colleagues^21^:

"*NCD public policies are broad statements made by government of goals, objectives and means in order to create a framework for activity directed at the prevention and control of NCDs. Such statements may be written or unwritten, explicit, or implicit*."

In order to guide our data collection and analysis on identifying factors promoting and hindering policy development and implementation, we reviewed theories of the policy process.^8,9^ We chose the multiple streams framework (MSF)^22^ as one that has been found useful in examining health policy agenda-setting, formulation and implementation in other settings,^23-25^ including in LMIcs.^26-29^ Our account of the MSF is largely based on those given by Zahariadis^30^ and Cairney,^8^ and we considered five main elements, summarised in Box 1. Briefly these five elements are: the problem – how the issue of NCDs is defined, discussed, and framed; the policies – that have been adopted, developed, and implemented; politics – how this influenced and was influenced by the problem of NCDs; policy windows – particular times/opportunities when problem, policies, and politics come together and enable change. Finally, we considered the roles of ‘policy entrepreneurs,’ key individuals or groups who work to bring problems, policies, and politics together in order to open up policy windows. The data collection took place from May to September 2013.

**Box 1. Core Elements of the MSFa as Used in This Study**

- The problem – how the issue of NCDs, including their prevention and control, is defined, discussed, and framed
- The policies – where they are from, how they were developed and implemented
- Politics – how the issue of NCDs has influenced and been addressed by policy-makers
- Policy windows – particular times/opportunities when problem, policies and politics came together to enable/ generate change
- Policy entrepreneurs – key individuals who help to shape and bring together problem, policies and politics to create policy windows

^a^Based on the accounts in Zachariadis^30^ and Cairney.^8^ Abbreviations: NCD, non-communicable disease; MSF, multiple streams framework.

### Document Review

Documents were identified through the key informants, prior to or at the end of each interview, as well as from the Ministry of Health (MoH) of Barbados and Pan American Health Organization (PAHO). The documents are listed in Box 2. Each document was examined for ‘policy statements’ (stated goals, objectives or means – however, specific or general). The policy statements were allocated to one of the following categories: risk factor reduction: tobacco smoking, unhealthy diet, physical inactivity, alcohol consumption; health promotion and education; integrated healthcare for those living with or at risk of NCDs; and NCD surveillance, monitoring and research. In addition, documents were examined for any statements on establishing overarching structures, processes and funding arrangements relevant to NCD prevention and control, such as the establishment of a multi-sectoral NCD commission and the establishment of the Barbados National Registry (BNR). A spreadsheet-based data abstraction form was created, into which policy statements, as defined above, were abstracted. For each policy statement available details were recorded on responsible persons, targets, and resources.

**Box 2. Policy Documents Identified and Analysed for Policy Statements (Year, Source)**

A. Barbados Strategic Health Plan for Health 2002–2012 (2003, MoH, Government of Barbados)
B. Strategy for the Prevention and Control of Chronic NCDs (2004, MoH, Government of Barbados)
C. Healthy Hearts for Life - Report of the Task Force on the Development of Cardiovascular Services (2007, MoH, Government of Barbados)
D. Declaration of the Port of Spain: Uniting to Stop the Epidemic of Chronic NCDs (2007, CARICOM Secretariat)
E. Strategic Plan 2009-2012 for the National Chronic NCD Commission (2008, MoH, Government of Barbados)

Abbreviations: NCD, non-communicable disease; MoH, Ministry of Health.

### Key Informant Interviews

Participants for the key informant interviews (N = 25) were purposefully sampled for their involvement with NCD policy planning, implementation or evaluation in Barbados within and outside the health sector (see Table 1). Initial key informants included the Chief Medical Officer, the MoH NCD focal point, and the chair and members of the existing multi-sectoral NCD Commission. The Commission, which though appointed by the Minister of Health and including some MoH officers as ex-officio members, is otherwise comprised of members who are not Government employees and it operates independent of the MoH, while aligning its interventions and activities in support of the Ministry. Additional key informants were identified through a stakeholder analysis and ‘cascading’ (ie, suggestions arising from informants already interviewed). Semi-structured interviews were conducted with 11 key informants from the MoH to provide a narrative on the process of formulating MoH-led NCD policies, and insights and experiences on the current state of implementation of these. Further interviews with 14 key informants outside the MoH were used to provide an outsider perspective, as these were informants who are and have been involved in the multi-sectoral effort of implementation. All semi-structured key informant interviews took place at a location convenient to the interviewees (for most, their place of work); interviews lasted 30-90 minutes and were voice recorded and transcribed verbatim. A copy of the interview guide is provided in online Appendix A (Additinal file). The structure of the guide was informed by the major categories used in the PAHO Strategic Plan of Action for NCDs, and was flexibly used as appropriate to the role of the key informant.

**Table 1:** Number of Key Informant Interviews by Sector

**Sector**	**Number**
MoH (including high level medical officers, representatives of drug service, government hospital, NCD Commission)	11
Other Government (including education, agriculture, government information service)	3
Civil society (including senior academics, leaders of health NGOs, representatives of trade union, faith-based organisation)	8
Private (including industry, health insurance, private health provider)	3
Total	25

Abbreviations: NCD, non-communicable disease; MoH, Ministry of Health.

### Data Analysis

The technique of framework analysis, a pragmatic approach which is explicitly geared towards using qualitative data collection to inform policy and practice,^31^ was used to analyse the key informant interviews. After familiarisation with the data, a thematic analysis was undertaken to develop a coding scheme and a framework table developed to index all interview content. The coding scheme analysis incorporated key aspects of the MSF. This interpretive analysis aimed to explain successes and challenges in policy formulation through the concepts of problem stream, politics stream, policy stream, policy windows and policy entrepreneurs. The coding process was aided with the qualitative analysis software Dedoose (version 4.5; http://www.dedoose.com/) that enabled themes to be collated and compared across interviews. The data collection and reporting adheres to international guidelines on qualitative research (such as the Biomed Central review guidelines for qualitative research - http://old.biomedcentral.com/authors/rats). All quotes were selected when they illustrated a common viewpoint, or if they provided unique information from a particular sector. Each quote is attributed to either a member of the MoH, the private sector or a civil society organisation. The names and positions of the individuals quoted are not given in order to protect their identity.

The contents of all the documents reviewed were summarised and mapped to the recommendations of both the 2011 PAHO/ CARICOM Strategic Plan of Action on NCDs^4^ and those of the WHO.^5^ This included identifying any possible inconsistencies between the documents. From this a ‘gap analysis’ against current PAHO/CARICOM/WHO recommendations was performed.

Coding of the key informant interviews and abstraction from the policy documents were checked at the time of the analysis and interpretation of the data. This involved meetings between the Barbados-based researchers, who discussed interpretation of the findings and went back to the original data to confirm, or refute, their initial conclusions. Judgements on the implementation of identified policy statements were made based on the reports given by key informants during their interviews. There were no formal evaluations of NCD policy to draw upon, with this current study representing the most structured evaluation to date.

## Results

Twenty-five key informant interviews were conducted, transcribed and analysed. Key informants were from the MoH, three other government departments (education, agriculture, and government information service), civil society, and the private sector (Table 1). Out of 17 documents identified and reviewed, 5 (listed in Box 2) were core policy documents and underwent detailed analysis. A full list of all 17 documents that were identified and reviewed is given in online Appendix B (Additinal file). Policy statements, as concurrent at the time of the study, from these 5 documents and comments on their implementation (based on progress, or lack of it, as reported by the key informants) are summarised in Table 2.

**Table 2:** Summary of Stated Policies on NCDs in Barbados *at the Time of the Study*, Their Source Document (See Box 2 for Key), and Comments on Their Implementation From Key Informant Interview Data

**Summary of Stated Policies**	**Source**	**Comments on Implementation**
**Overarching Structures, Processes and Finance**		
1.1 Establish national multi-sectoral NCD Commission	B	1.1 Barbados NCD Commission established March 2007 (predated Port of Spain Declaration, document D). Multi-sectoral membership, 10 meeting/year. Dedicated staff and budget.
1.2 Establish post of SMO of Health for NCDs		1.2 SMO NCDs established. Not all support staff in place.
1.3 Establish Health Promotion Unit		1.3 Health Promotion Unit established with Senior HP Officer and 1 HP Officer (not 2 as planned).
1.4 Establish Committee of Focal Points from other Ministries		1.4 No functional Committee of Focal Points from other Ministries.
2.1 Use revenue from tobacco, alcohol and other products to support the work of the NCD Commission	D	2.1 Ministry of Finance has declined to ear-mark tobacco or alcohol taxes to support the Commission, instead giving annual subventions for its operations, through the MoH.
**Risk Factor Reduction**		
Tobacco		
3.1 Pursue immediately a legislative agenda for passage of the legal provisions related to the FCTC	D	3.1 FCTC ratified in 2005.
3.2 Legislation to limit or eliminate smoking in public places		3.2 Legislation prohibiting smoking in all indoor spaces enacted and fully implemented in 2010.
3.3 Ban the sale, advertising and promotion of tobacco products to children		3.3 No sale, advertising, or promotion to children. Partial ban in place through “gentlemen’s agreement” but not legislated.
3.4 Effective warning labels		3.4 Implementation of warning labels failed as regional initiative, but a regional standard has been established for each country to implement. Progress is sporadic, and not implemented in Barbados.
3.5 Introduce such fiscal measures as will reduce accessibility of tobacco		3.5 In 2011, taxes comprised 48% of tobacco sale price (PAHO Barbados Tobacco Control Report 2011).
Alcohol		
3.1 Behavioural intervention programmes…to address*..*.alcohol abuse prevention	B,C	Limited, general, mention of alcohol. Reluctance nationally and regionally to address alcohol-related harm, because of economic and perceived cultural importance. No evidence further development or implementation.
3.2 Promotion of moderate alcohol consumption		
Diet		
4.1 Creation of a ‘National Food Authority’	B, E	All are statements that were made without specific actions for their further development or implementation, and there was no evidence from the interviews that significant progress had been made in these areas.
4.2 Ensuring that only healthy foods and snacks available in schools and healthy options are available at work places		
4.3 Develop incentive/recognition programme for vendors/restaurants to offer healthy options		
4.4 Promote ‘backyard gardens’		
4.5 Reduction in high fat, sugar and salt intake, and increase fresh fruit and vegetables		
5.1 Education sectors to promote programmes for healthy school meals	D	5.1 Dietary guidance produced for school meals, however, this is against a backdrop of aggressive marketing of unhealthy foods to school children, including branding of classroom items with local fast food logos.
5.2 Support elimination of transfats		5.2-4 Seen as requiring regional action. CFNI was to be the regional focal point. However, CFNI now merged into Caribbean Public Health Agency with diminished capacity. Requires regional negotiating machinery to pursue fair trade policies, and no evidence of progress at the time of the study.
5.3 Support for mandating the labelling of foods		
5.4 Promote greater use of indigenous agricultural products and foods		
Physical Activity		
6.1 Ensure physical activity is part of curriculum for every child	B, D, E	6.1-2 No evidence of implementation.
6.2 Re-introduction of physical education in our schools where necessary		
6.3 Provide opportunities and facilities for physical activity at work		6.3 Reported that some uptake by the private sector of Workplace Wellness programmes.
6.4 Promote policies and actions aimed at increasing physical activity in the entire population eg, through worksites, through sports, especially mass activities		6.4 Some mass media awareness raising from the MoH, such as around Caribbean Wellness day (see next section).
6.5 Commit to increasing adequate public facilities such as parks and other recreational spaces		6.5 Urban planning department has commitment to providing public space for recreation in new housing developments.
**Health Promotion and Education**		
7.1 Develop education programmes and campaigns providing information about NCDs, and in support of wellness, healthy life style, and improve self-management of NCDs	B, D, E	7.1 Regular mass media activities by the Health Promotion Unit, such as campaign on salt reduction.
7.2 Create a reward-based system to encourage participation in the Healthy Schools Initiative		7.2 No evidence of implementation.
7.3 Embrace the role of the media as a responsible partner in all our efforts to prevent and control NCDs		7.3 Sub-optimal media engagement and public education, due in part to lack of funding. Compared to funding for media for HIV/AIDS, NCDs have had no funding and relatively little media.
7.4 Celebrate second Saturday in September as ‘Caribbean Wellness Day’		7.4 Caribbean Wellness Day celebrated annually since 2008. Has not been used to sufficiently to promote activities across sectors. However, impression is that frequency of organised physical activity events has increased.
7.5 Increase work wellness programmes		7.5 Reported that some uptake by private sector.
7.6 Support faith-based health promotion		7.6 Little evidence of implementation.
**Integrated Disease Management**		
8.1 Establish by mid-2008 comprehensive plans for the screening and management of chronic diseases and risk factors so that by 2012, 80% of people with NCDs would receive quality care and have access to preventive education based on regional guidelines	C, D, E	8.1-2 Regional treatment guidelines disseminated but uptake and impact unknown; lack of systematic monitoring on coverage and disease control.
8.2 Comprehensive plans for screening and management of NCDs		
8.3 Establish NCD clinics within government primary healthcare system		8.3 Clinics for diabetes and hypertension within government primary healthcare; drugs for diabetes and hypertension available free of cost.
8.4 Annual training to primary healthcare professionals on use of protocols		8.4 Some training workshops in the use of the regional guidelines have occurred, not annual training. However, since Jan 2013 medical practitioners need to show evidence annually of continuing medical education to remain on the medical register.
**Surveillance, Evaluation and Research**		
9.1 Develop comprehensive health information strategy	C, E	9.1 Work still in progress on developing health information system. Lack routine data on disease treatment and control.
9.2 Establish cardiac and stroke event registry		9.2 Barbados National Register of Strokes and Myocardial Infarctions established, including data from private sector, but relative lack of resources to produce timely reports and investigate findings.
9.3 Commission research to develop appropriate baseline measures for health improvement and service framework		9.3 Partially met through 9.2, and through supporting MoH personnel to undertake research as part of studying for a master in public health.
9.4 Repeat the Behavioural Risk Factor Survey in 2010		9.4 Repeat of risk factor survey being completed at the time of the study.
9.5 Private sector data fed into national NCD data		9.5 Inadequate routine private sector data.

Abbreviations: SMO, Senior Medical Officer; FCTC, framework convention of tobacco control; CFNI, Caribbean Food and Nutrition Institute; NCD, non-communicable disease; MoH, Ministry of Health; PAHO, Pan American Health Organizatio

We start by describing a ‘policy window’ that opened in 2005 and the factors that led to it opening at that time. We then go on to look at progress from since that window opened, up to September 2013, examining the ongoing problem description and definition, policies, politics and the role of policy entrepreneurs. We describe the findings from the study on the processes of policy agenda-setting, formulation and implementation using the broad headings of the MSF. The description of the opening of the policy window in 2005 might be seen as the first agenda-setting in response to the overall problem of NCDs, and the description of what follows (in terms of problem, policies and politics) as ongoing grappling with the development and implementation of policies. However, it was clear from the reports of key informants that the process played out more iteratively, and this is reflected in the accounts that follow.

### Opening of a Significant Policy Window From 2005 to 2007

The year 2005 was identified as a pivot point in the adoption of significant government policy to address NCDs. Several factors seem to have contributed towards this. Firstly, there was increasing awareness at the highest level of government of the problem of NCDs. In 2001, there was the Nassau Declaration, made by the CARICOM Heads of Government, ‘The Health of the Region is the Wealth of Region.’^32^ Although HIV/AIDS received the greatest prominence in this declaration, it also committed to the development of a regional strategy for the prevention and control of NCDs. Within Barbados, the work of the Chronic Disease Research Centre (CDRC), part of the University of the West Indies, was documenting the high burden of NCDs, which found regular outlet in the Barbados national media. The work of CDRC was regularly communicated to the MoH, and through two senior and highly respected health professionals was directly communicated to Ministers of Government, including the Prime-Minister of the time, and other senior politicians.

It was this combination of increasing awareness of the problem and political engagement that led to a request for the MoH to produce in 2004 a strategy on NCD prevention and control (document B in Box 2). The recommendations in this document were reinforced by a meeting of international experts in Barbados in 2005, which was hosted by the Barbados MoH, the Caribbean Office of the PAHO, and the University of the West Indies. It was chaired by a highly respected Barbadian Dean of Medicine at the University of the West Indies.

A key outcome of these processes of increasing awareness of the problem of NCDs, political engagement by key health professionals (policy entrepreneurs), and specific policy recommendations was that the Cabinet of the Government agreed to fund three new posts: a senior medical officer of health, devoted to NCDs, and two health promotion officers (Table 2, 1.2-1.3). In addition, it was agreed to establish and support a multi-sectoral national NCD Commission (Table 2, 1.1), which first met in early 2007.

*“So my involvement started, I think it was 2005, when there was a consultation held here that was really driven in many respects [by …] the [then] Chief Medical Officer…and it brought together persons from the international community to discuss the chronic diseases.…Arising out of that, was a decision among others to establish a chronic diseases commission…”* [4, MoH].

Barbados helped to lead the way in CARICOM (along with Bermuda, which had established a ‘Well Bermuda Partnership” in 2005), with all CARICOM members committing later in the year to the establishment of multi-sectoral national NCD Commissions in the Port of Spain NCD Declaration of September 2007.^7^

### Problem: High Level of Awareness but Marked Divergence on How It Is Framed

There was some consistency but also considerable divergence in how key informants described the problem of NCDs, and its required solutions, in Barbados. As described above, NCDs had been identified as a major health burden in the region, and a threat to social and economic development. There was complete consistency that there was a high level of awareness, from the general population through to Government, in the existence of NCDs as a major problem that needs to be addressed. This awareness could clearly be traced to the effective knowledge exchange between local, regional, and international organisations, the MoH and the media.

*“…Barbadians are very concerned about the effects of chronic diseases and I think that is because we’ve done a very good job in telling them that these things cost a lot of money and they are a major portion of our budget and so people understand the cost of ill health”* [9, MoH].

It was widely perceived that the NCD burden threatened the development and prosperity of the country, was experienced personally by many, and had entered the public conscious. There was a divergence, however, on how the problem was framed and consequently on the best way of tackling it. In particular, civil society and private sector respondents, but also some MoH personnel, tended to frame the issue as one almost entirely of personal responsibility for unhealthy life styles.

*“…cause largely I think the problem is that people are really ignoring their own health issues, and it’s a means of how do we get that message over to people, to reconsider their lifestyles as it relates to their health and weight, food choices.”* [7, Private].

*“I mean, a lot of the barriers may just stem from the individuals themselves, so it’s […] up to the individual or up to […] us to try and empower the individual to do what is […] necessary in order to reduce the burden of chronic diseases…”* [14, MoH].

In a country that has developed rapidly since independence in 1966, with a large and growing middle class, high levels of NCDs were seen as being connected to unhealthy eating, including a liking for and reliance on imported ‘fast’ and processed foods, and high levels of physical inactivity, partly due to the use of motorised transport and the high status accorded to car ownership. Some informants framed this in terms of macro-social determinants – a car-centric infrastructure that discourages walking or cycling, and an over reliance on food imports – and saw a key role for Government and society to modify these unhealthy environments in order to reduce the burden of NCDs. However, there was also a strong belief expressed by some key informants that the real issue was not to do with the broader environment, but with individuals needing to take responsibility for their own health, and essentially to eat less and be more physically active. In addition, it was noted that emphasising personal responsibility was the more acceptable way to frame the problem for politicians, thus, removing the need for legislative measures. In other words, if challenging the acceptance and behaviour of fast food outlets could prove unpopular and politically difficult, educating the population about the dangers of frequenting these places might be the easier solution.

*“[…] there is a lot of scope in there to put policies in place. But you know, […] I get the sense that […] our politicians almost sort of instinctively prefer to bring about change without using legislation”* [4, MoH].

*“…a lot of persons especially at the policy level feel that its individual people’s responsibility. They do not understand that policy level facilitation is necessary to get the environment that allows people to make the right choices for all of the risk factors”* [9, MoH].

However, as illustrated by the quote immediately above and those that follow, several key informants did emphasise the need for environmental changes to help support individuals to change behaviours.

*“We need policies around types of activity. Whether it be, as I said, activities around personal transportation, whether it be policies around recreation, so government policies around making gyms more affordable […]”* [4, MoH].

*“…people must take responsibility for their eating and their exercising. Yes that is true. But that alone is not going to cut it, and there is no evidence to that alone cuts it”* [11, Private].

In summary, the problem of NCDs was seen as part of the package of Barbados’s economic development and linked to aspirations of its population for ‘western’ lifestyles. However, at one end of the spectrum the problem causing NCDs was framed almost exclusively as one of individuals not taking responsibility for their own health, and at the other end of the spectrum it was seen as being due to broader environmental factors (eg, built environment, food environment).

### Policies: Success Where There Is Clear External Guidance and the Issue Is Politically Uncontentious

Policies at the time the study was conducted, six years on from the ‘policy window’ described above, are summarised in Table 2. Policies formulated in the Port of Spain Declaration, that were separately developed and implemented in Barbados, were the establishment of designated NCD posts and of a multi-sectoral NCD Commission. Funding for the posts established in the MoH, as agreed in 2005, was continued despite a change in Government in 2008 and the global economic downturn since 2008. However, it was noted that not all the posts originally promised were funded (Table 2), and that compared to funding for HIV/AIDS funding for NCDs was disproportionately low.

*“…in 2005 within Barbados it seemed as if the time was right, it was, it has never been easy but it was relatively smooth passage to have the post(s) created, we didn’t get all the post(s) we wanted and that is why NCDs is really a skeleton programme as compared to HIV”* [9, MoH].

This difference was partly attributed to the fact that HIV/AIDS received substantial external donor funding, whereas funding for NCDs was predominantly from the Government of Barbados.

Another policy statement that was clearly implemented is the multi-sectoral national NCD Commission. It has met regularly since its inception, with financial support (for organisation of the meetings and for some projects) and secretariat support from the MoH and under the continuing leadership of one of the highly respected individuals who helped get NCDs onto the political agenda in 2005.

*“I think things went well in Barbados because we had early buy in from key stakeholders, and that is very critical…, civil society, you know, public sector, private sector, policy-makers, we got them all on-board early…”* [16, MoH].

The clearest additional policy success since 2007 was the ratification of the framework convention on tobacco control (FCTC) and implementation of some of its articles (Table 2), including banning smoking in public places. Part of the reason for the successes in tobacco control was that it came with clear guidance on the policies to implement and with a relatively low prevalence of smoking it was politically uncontentious.

*“…tobacco has the advantage of the FCTC [framework convention on tobacco control] which is this international framework, and therefore, very clear guidelines on what to do, how to do it, when to do it etc. and you sign the treaty and you have obligations that by this date you have to do this thing and so on and so forth. Well-organised. And so there, even though it is not a priority in terms of the burden of tobacco use in Barbados, it’s less than 10% for men and less than 2% for women, still because the systems are there, it facilitates advances”* [2, Civil Society].

*“…the Port of Spain Declaration mandates that countries implement the FCTC…as well as the [United Nations] high level meeting…urged countries to implement the FCTC and…here in Barbados we have been working on a ban on smoking in public places, it preceded the FCTC, we’d been working on it for a number of years...”* [8, MoH].

It was noted that discussions around the FCTC were also important in helping to demonstrate to policy-makers that there were viable options for structured policy responses to NCDs.

Another success, led by the Health Promotion Unit, has been the celebration of Caribbean Wellness Day, which was mandated in the 2007 Port of Spain Declaration^7^ as the second Saturday in September. Again, this was facilitated by clear guidance and supports from the regional Caribbean level, with regional branding, information and guidance facilitated by PAHO. In Barbados, multi-sectoral involvement was encouraged and facilitated by private sector and civil society membership of the NCD commission.

An example of policy transfer that has been less successful is the implementation of regional guidelines on the treatment of NCDs. The issue here is that while regional clinical guidelines have been produced and adopted in Barbados, there is not a clear pathway to successfully implementing those guidelines or auditing their impact.

*“Well, the Caribbean Health Research Council…has guidelines on treatment of diabetes and hypertension, asthma I think, are the main guidelines... And I believe that Barbados has adopted these guidelines. The issues is, so the policies set, the issues, the implementation of the policy, I think that is where the challenges still remain…”* [2, Civil Society].

*“A few years ago, I think through PAHO, we set policy for treating diabetes.…So it was something that was done, and it was not acted upon. So there are lots of policies made that are not acted upon. I don’t know where the paperwork gets lost”* [10, Civil Society].

In some areas there were examples of policy statements that lack specificity and for which there was little evidence of action. Perhaps the clearest example of this is the lack of effective policy on the harm caused by alcohol, where the only statements were very broad (Table 2), with no evidence of any implementation. Reasons for this are largely political, and discussed under the politics heading below.

### Politics: High Level Attention but Working in Silos

There was consensus that NCDs have and continue to receive high level political attention, with Barbados being seen as a leader in the region in areas such as the provision of free medication for those with NCDs, and Government support for surveillance, such as the BNR, which covers heart attack, stroke and cancers.^33^

*“I think that political support at the high level is very important in making it a priority and pushing it. It’s not always financial resources that you need […]”* [8, MoH].

*“Government has really been very good. I think the Barbados Government, has been among the leaders.…I mean the idea that, you know, you have the medication free at the point of contact. The idea that they have provided health centres, the idea that they have had education in health centres for diabetes, that’s all good”* [22, Civil Society].

Despite this political leadership role within the Caribbean region, informants noted that domestically some political priorities, nonetheless, competed directly with NCD prevention and control, with the most obvious example being prevention of alcohol-related harm. Anything that might be seen to damage alcohol production (beer and rum are produced locally), revenue or export was perceived as politically unsupportable.

*“As far as the alcohol is concerned that is another story. (laughter) We make it, we have to sell it, so I guess they try to figure out ways, to encourage people not to over-indulge”* [10, Civil Society].

*“We haven’t touched alcohol, that’s a ticklish thing - I mean our country is an alcohol country, we produce alcohol”* [24, MoH].

Key informants also described a large fast food sector, with both local and well-known trans-national fast food chains receiving political support to increase their activities on the grounds of promoting economic growth. This links to the problem definition, described earlier; while the NCD burden was politically recognised as a threat to social and economic development, framing the problem as one of personal responsibility means that it is not in direct conflict with political support for fast food businesses.

However, even if a structural approach to changing environments was accepted, the political landscape of ‘silo-working’ did not support this endeavour. Thus, despite the high level political attention that NCDs have received, there was frustration expressed that within Government the response is almost exclusively through the MoH, with very limited success in engaging other Ministries such as education, trade, agriculture, or finance (this is illustrated in the 3 quotes at the end of the section on problem definition). As an example, MoH strategic plans included nutrition and physical activity policy recommendations for the Ministry of Education; however, this would mean setting a corresponding policy agenda, and then formulating and implementing such policies, within the Ministry of Education and their own budget.

Similar frustrations were expressed in terms of collaborating with other sectors of society, such as civil society organisations and the private sector with their own processes of agenda-setting and subsequent action:

*“…finding the best way to work with other sectors is… challenging. Because as I said before they have their own agenda and then when you come trying to do something they …see it as additional, so we need to find a better way of engaging them and sustaining the work”* [8, MoH].

*“…I think people talk to each other, but I don’t think we are collaborating nearly as much as we should, and I mean we still suffer from this silo thing, you know ‘This is mine’ and ‘I want to get recognition for this. So I don’t want you to share this’...”* [22, Civil Society].

If an ‘all of society’ approach was to be pursued, civil society key informants expressed the view that political leadership, from government, is required:

*“If government doesn’t engage nothing moves. We were able to put bans on cigarette smoking in public places because a Minister of Health made that one of his defining dictates, so it happened. Unless an agricultural minister says you have to grow more food or else, you will never grow more food”* [1, Civil Society].

### Policy Entrepreneurs: Crucial Influence of Respected Individuals With High Level Political Connections

Much of the success that Barbados has had since 2000 in raising awareness about NCDs and initiating policy responses was attributed to the advocacy of a small number of highly respected individuals – policy entrepreneurs. These are senior medical and public health professionals, including researchers in defining the burden of NCDs, and, they had the credibility to gain access to senior Government members, including the Prime Minister and Minister of Health of the day. This was still seen to be the case since the inception of the national NCD Commission in 2007, with the professional credibility and political connectedness of the Chair being crucial.

*“[…] having the Commission has made a lot of difference because that gives it a national persona and then having someone like [the Commission Chair] to chair it also increases the credibility and the visibility to keep moving forward”* [8, MoH].

*“My concern is that if [current identified policy entrepreneur] isn’t around for whatever reason […] I think everything would cease and I think that is poor succession planning but also speaks to the fact you know, the hard work that he clearly does to try and bring this together, we all seem to work very independently”* [6, Civil Society].

These well-recognised and connected (in a generic political sense, not party political) public health advocates have played a key role in putting and keeping NCDs on the political agenda. Their role seems to have been crucial in gaining political commitment, including the ongoing funding of new posts in the MoH and the establishment and support for a national NCD Commission and the NCD research agenda. Moreover, the role of policy entrepreneurs and their long-standing status within Barbadian society seems also to have been important in ensuring that commitments made by one government continued when the government changed in 2008.

## Discussion

### Summary of the Main Findings

This study has documented the considerable progress that had been made from 2000 to 2013 in formulating, adopting and implementing policy on NCD prevention and control in Barbados. Clear successes included the creation of new posts within the MoH, the establishment of a national NCD Commission with membership from government, civil society, the media and the private sector, support for active NCD surveillance and the implementation of some aspects of the FCTC. Analysis of the findings through the lens of the MSF suggests that key to these achievements was the combination of widespread awareness about the burden of NCDs, due in part to research conducted in Barbados by the University of the West Indies, and the activities of a small number of politically well-recognised and connected ‘policy entrepreneurs,’ who were able to suggest policy solutions, to the Government, including the need for funding. It is important to note that these processes of policy agenda-setting, formulation and implementation could not easily be disentangled into these separate stages of policy-making. The boundaries between these areas were often blurred and many policy initiatives did not clearly follow this sequence. As an example, the regional Port of Spain Declaration could be seen as both actual policy formulation as well as agenda-setting for the separate nations including Barbados. Implementation of some of these policies such as the national NCD Commission, was intended to establish a body to push forward further policy formulation and implementation.

The study has also highlighted areas where progress was limited. A general shortcoming was the lack of inter-sectoral action, despite acknowledgement of it in policy statements. A lack of an effective ‘whole of government’ approach was particularly noted, where ministries outside of health, including finance, trade and urban planning, were little engaged in policy for the prevention and control of NCDs. Factors that appeared to be important in this, are that these non-health ministries viewed NCDs as a health issue and therefore, not one for their concern and their already limited budgets. In addition, many key informants saw the problem of NCDs as being largely one of personal responsibility ie, people need to be given information about healthy behaviours and encouraged to act responsibly. The need for broader environmental changes to support healthier behaviours was emphasised by only some of the officials in the MoH. The problem was, therefore, not one on which there was sufficient consensus on what should be done, it was not a ‘valence issue.’^8^ In addition, it was noted that politically it is easier to call for personal responsibility than to seek changes in regulatory, legislative or fiscal measures.

### Context of Other Research

This study adds to a small but growing body of research that is concerned with the process of health policy-making for the prevention and control of NCDs. The study is timely, given the emphasis on policy interventions in the 2011 UN high level meeting on NCDs, an emphasis reflected in the WHO Global Action Plan on NCDs.^5^ The study is consistent with a large body of interdisciplinary work on policy analysis that rejects simple linear understandings of the policy process towards exploring the complexity, messiness, and dynamic nature of policy-making.^9^ Within the field of health policy, it had been noted by Walt and colleagues that there was a need to move beyond simple description to applying theory to assist in understanding the policy process.^34^

A number of other studies of health policy have made use of the MSF as part of a systematic analysis of different factors and influences at play. Examples, include public health policy to promote walking in England,^35^ and public health policy to improve diabetes care in Ireland.^36^ These have highlighted the fragility of some policy windows that instigate attention and action – such as the Olympic Games to create traction for population-level physical activity strategies^35^ – and the importance of influential and committed policy entrepreneurs who are required to create or capitalise on such windows.

There is limited published experience in using the MSF for health policy analyses in low- and middle-income regions. The utility of the MSF as a health policy analysis tool in a low-income setting, Burkina Faso, was evaluated by Ridde.^29^ He found that it was useful analytical framework to examine factors supporting and hindering three broad stages of the policy process: agenda-setting, formulation and implementation. In this study, we have also found the MSF useful to examine causal factors across these aspects of the policy process. Other examples of the MSF being used in LMICs or regions include its use in examining policy initiatives in Iran^27,37^; the failure of a health insurance system in Lebanon^38^; the introduction of a national health insurance scheme in Ghana^26^; and the development of maternal health policy in India.^28^ These studies can serve to broaden our understanding of the policy process – and the utility of MSF as an analytical tool – by drawing out the complexity in different settings and contexts and their influence on policy-making. For example, as we also found in our study, in political systems with small populations, policy entrepreneurs may hold a very particular, and perhaps distinctly influential, role.^39^ In our study, we could also explore in greater depth, in what way national policy-making is influenced by regional and international agenda-setting and policy-making, in particular the importance of policy transfer.

Our paper, therefore, adds to a relatively small literature on applying the MSF to policy-making relevant to the prevention and control of NCDs, and as far as we are aware is the first to do so in a developing country setting.

### Implications for Non-communicable Disease Policy Development and Implementation

What lessons might be tentatively drawn from this study for improving the development and implementation of policy on NCDs in similar settings? In answering this question, we take the view point not of policy-makers, but of someone wishing to influence the decisions made by policy-makers, such as public health practitioners.

#### Problem: Moving Towards a Structural Response

The first lesson we draw concerns the importance of how the problem is defined and framed. It is widely accepted within public health that the determinants of behavioural risk factors for NCDs, including physical inactivity, unhealthy diet, tobacco smoking, and excess alcohol consumption are embedded within the structure of modern societies – the social determinants of health.^10,40^ Changing behaviour requires much more than simply appealing to ‘personal responsibility,’ and must include changing the environments (eg, physical, fiscal, social, cultural, information) within which choices are made.^5^ A recent systematic review of the public acceptability of government interventions to change health-related behaviours suggests, not surprisingly, that greatest support is present for those that are least intrusive, but which are often the least effective.^41^ Working to increase the public acceptability of, and ideally the public demand for, effective government policy measures is essential to enable policy-makers to implement them.^42^ In short, there is a need to frame the problem of NCDs beyond one of personal responsibility, and to do this not only with policy-makers but with the wider public whose support for policy changes is essential.

#### Policies: Enabling Policy Transfer

Another lesson concerns the availability of policies that can be readily transferred and adapted. Of policies addressing the major NCD behavioural risk factors, by far the greatest success was in addressing tobacco, with ratification of the FCTC and implementation of some of its articles including legislation on smoke free public places. Part of this success relates to the public acceptability of antismoking measures, given the low prevalence of tobacco smoking in Barbados (in 2007 daily tobacco smoking was estimated at 8.4%; 15.3% in males, 2.2% in females)^16^ and lack of commercial tobacco production on the island. However, it also relates to the fact that FCTC and its articles provide clear guidance on what should be enacted and implemented across different sectors. The availability of ‘readymade’ policy is particularly useful in settings where there is limited human resource capacity to draft evidence-based policy de novo, as is typically the case in small island developing states and in many larger LMICs.^43^ The WHO Global Action Plan on NCDs contains a long list of ‘evidence-based’ policy recommendations,^5^ which tend to be general statements, such as on diet and physical activity, that require much additional formulation to become effective, implementable, policy measures. Governments could be greatly assisted by the availability of detailed policy statements, with guidance on implementation, which could be transferred and adapted to their setting. Regional organisations, working on behalf of individual governments, could take the lead in developing such statements, or sharing such policies across countries from those who have already developed them, to those still contemplating. In the Caribbean that could be facilitated by the Caribbean Public Health Agency and the PAHO.

#### Politics: The Importance of Multi-sectoral Cooperation and Influence of Policy Entrepreneurs

The main lesson under the heading of ‘politics’ is from the challenge of involving government ministries other than health in the response to NCDs. The national NCD Commission was reported as providing a successful vehicle for engaging with leaders in civil society organisations and the private sector, but cannot be the vehicle for engaging different government ministries such as education and agriculture in a ‘whole of government’ response. It has been noted elsewhere that the mechanisms required for cooperation between sectors within government are different to those required for cooperation between different sectors of society.^44^ In recognition of these differences, it is suggested that multi-sectoral responses be considered in two arenas – with the term ‘whole of government’ used to describe ‘cooperation among agencies of government,’ and ‘whole of society’ used to describe cooperation between the three main sectors of the state (government, private, and civil society).

Mechanisms for ‘whole of government’ inter-sectorality include making the issue one that is regularly on the agenda of cabinet meetings, the establishment of inter-ministerial and parliamentary committees and joint budgeting between ministries.^45^

Finally, a crucial mechanism for multi-sectoral working, seems to be Kingdon’s policy entrepreneurs. The policy entrepreneurs in this study held a crucial position and ability to initiate and facilitate such multi-sectorality as they had influence in various sectors. Our findings also suggest that the influence of policy entrepreneurs could be even greater if the problem of NCDs was framed more in public debate as one requiring government driven environmental change, in addition to appealing to personal responsibility, and with the greater availability of well-articulated policy options that could be adopted in Barbados, as was the case with the FCTC.

## Strengths and Limitations

A strength of this study is that a broad range of stakeholders were interviewed to gain insight on what had been achieved and why, getting beyond official accounts in documents and from members of government. A potential weakness of going for a broad range of stakeholders is that some sectors were less well-represented. For example, while leaders of most national health non-governmental organisations (NGOs) were included, operational staff or non-health NGOs such as faith-based organisations were less well-represented. We justified this decision with our focus on Government-led policy which orientated the sample towards participants with direct involvement or insight into health. A strength is that official policy documents were scrutinised in detail, and a theoretical framework was used to investigate the policy process.

We chose the MSF as one that has proven useful in examining health policy in other settings.^23-25^ A recent review of the uses of the MSF across all fields of study defined 7 categories of application.^39^ Our application falls within the category in which the use of MSF is ‘to structure and help explain policy change in a detailed case study.’^39^ We have not attempted to appraise the adequacy of, or develop the content of, the MSF, nor did we use other theoretical frameworks. Applying another theoretical framework, such as the advocacy coalition framework,^46^ might have provided additional or even different insights. A further limitation is that our study has not examined the health outcomes that the policy is intended to influence. Determining whether trends in health outcomes are related to success in policy implementation would require a different type of study.

Finally, this study was undertaken in one setting, Barbados, and it cannot be assumed that the lessons drawn here are generalisable to other settings. Rather our interpretation of causal relationships in this study should be seen as hypotheses that must be subject to further critical evaluation.

## Conclusion

The broad aim of this work, undertaken in Barbados, was to contribute to an overall goal of understanding the successes and difficulties of formulating and implementing policy on NCDs in the Caribbean and similar developing regions. Using the lens of the MSF we have identified: that NCDs are widely perceived as a major burden but that there is inconsistency in how the problem is framed, from one that is almost exclusively seen as an issue of personal responsibility to one requiring structural and environmental changes; the utility of well-formulated policy statements/programmes available for transfer and adaptation locally, such as the FCTC; and the key role played by highly respected local health professionals acting as policy entrepreneurs to raise awareness about the problem and promote political action.

Since the study was completed in late 2013, the MoH of Barbados in 2015 launched a new strategic plan for the prevention and control of NCDs. The study described here contributed to that, helping to highlight areas where greater specificity in policy statements, tied to measurable impacts and outcomes, were needed – such as on aspects of diet, physical inactivity, and alcohol-related harm. Barbados has also inaugurated an ‘Inter-Ministerial Task Force on NCDs’ chaired by the Minister of Health and tasked to oversee the ‘whole of government’ response to NCDs. In addition, Barbados has become the first country within CARICOM to enact, in June 2015, a tax on sugar sweetened beverages.^47^ This was a surprise to most observers as a proposal for this had been rejected a year earlier. How this tax came to be implemented, and its impact, will be the subject of further study.

In conclusion, this study has provided insight into policy-making around NCDs in the small island developing state of Barbados. We believe that the methods and findings from our study are useful to guiding and understanding policy-making in other developing countries and regions facing a high burden of NCDs.

## Acknowledgements

The study was funded by a grant from the PAHO, and we thank in particular Dr. Branka Legetic and Dr. Tomo Kanda for their support of the study. We thank all the key informants participating in the study, and for providing policy documents, and Ms. Lisa Bishop for transcription of the interviews. Finally, we thank Sir George Alleyne, Professor Anselm Hennis, and Dr. Madhuvanti Murphy for comments on earlier versions of this paper.

## Ethical issues

University of the West Indies and Barbados MoH Research Ethics Committee Institutional Review Board approved this study. Particular effort was made to ensure that none of the key informants, some in high governmental position, would be directly identifiable. For this reason names of the key informants are not given, only the organisations or areas they represent. All key informants were given an opportunity to review and comment on the detailed final report in which interview data were used.

## Competing interests

Authors declare that they have no competing interests.

## Authors’ contributions

NU, CG, AS, TH, and RB conceived and contributed to the design of the study; NU, CG, AS, and TH conducted the interviews. CG led the analysis of the key informant interviews, with input from NU, AS, and TH. NU led the analysis of the policy documents. CG and NU wrote the first draft of the paper. All authors contributed to and approved the final draft of the paper.

## Authors’ affiliations

^1^Chronic Disease Research Centre, University of the West Indies, Bridgetown, Barbados. ^2^MRC Epidemiology Unit and UKCRC Centre for Diet and Activity Research, University of Cambridge, Cambridge, UK. ^3^Healthy Caribbean Coalition, Bridgetown, Barbados. ^4^Prevention Research Center in St. Louis, Brown School, Washington University in St. Louis, St. Louis, MO, USA. ^5^Division of Public Health Sciences and Alvin J. Siteman Cancer Center, Department of Surgery, Washington University School of Medicine, Washington University in St. Louis, St. Louis, MO, USA.

## Additional Files

Additional file 1 contains Appendix A and B.

## Key messages

**Implications for policy makers**

- Public policies have a key role in the prevention and control non-communicable diseases (NCDs). There is little evidence from low- and middle-income or developing countries on factors promoting or hindering successful policy development and implementation for NCDs.
- A high level of public awareness on the burden of NCDs, supported in part through dissemination of local research findings, was key to putting and keeping NCDs on the political agenda.
- However, despite high levels of awareness on the burden, there was a widespread belief that reducing the burden of NCDs is largely a matter of encouraging individuals to take responsibility for their own health. There is a need for an informed public debate on the underlying environmental factors driving NCD risk in order to make some potentially highly effective legislative and fiscal measures politically possible.
- Well-worked up policy recommendations from international bodies, such as the framework convention on tobacco control (FCTC), that can be adapted locally can greatly facilitate policy formulation and development, especially when ‘in house’ capacity for policy development is limited. There is a need for similar, well-worked up guidance on areas such as nutrition and physical activity.
- Highly respected local champions, or policy entrepreneurs, can play a vital role both in shaping the debate and bringing together the need for a political response with locally appropriate policy initiatives.

**Implications for public**

Non-communicable diseases (NCDs), such as diabetes, cardiovascular disease, and cancers, are a growing burden in most developing countries. Government policy measures have a key role in reducing the risk of developing NCDs. Our study examines successes and challenges in developing and implementing policy in a developing country, Barbados, with a high burden of NCDs. The findings from this study should help to guide more successful development and implementation of government policy in this and similar settings. An example is that there is widespread perception of NCDs, shared by many policy-makers, that their prevention is predominantly about encouraging individuals to take more responsibility for their health. However, evidence suggests that at a population level this very difficult unless governments also take action to create health promoting environments. Respected and well-connected health professionals can be effective as ‘local champions,’ raising awareness within government about NCDs and the policy responses needed.

## References

[1] World Health Organization (WHO). Global status report on noncommunicable diseases 2010: description of the global burden of NCDs, their risk factors and determinants. Geneva: WHO; 2011.

[2] World Health Organization (WHO). Noncommunicable Diseases: country profiles 2011. Geneva: WHO; 2011.

[3] Pan American Health Organization (PAHO). Health situation in the Americas: basic indicators 2012. Washington DC: PAHO; 2012.

[4] Pan American Health Organization, Caribbean Community Secretariat. Strategic Plan of Action for the Prevention and Control of Non-Communicable Diseases for the Countries of the Caribbean Community 2011-15; 2011.

[5] World Health Organization (WHO). Global action plan for the prevention and control of noncommunicable diseases 2013-2020. Geneva: WHO; 2013.

[6] World Health Organization (WHO). United Nations high-level meeting on noncommunicable disease prevention and control. http://www.who.int/nmh/events/un_ncd_summit2011/en/index.html. Accessed January 25, 2014. Published 2011.

[7] Caribbean Community Secretariat. CARICOM Heads Adopt Declaration on NCDs. Declaraion of Port-of-Spain: Uniting to Stop the Epidemic of Chronic NCDs. http://www.caricom.org/jsp/pressreleases/pres212_07.jsp. Accessed January 25, 2014. Published 2007.

[8] Cairney P. Understanding Public Policy: Theories and Issues. Basingstoke: Palgrave McMillian; 2012.

[9] Sabatier P, ed. Theories of the Policy Process. 2nd ed. Cambridge MA: Westview Press; 2007.

[10] Committee on an Evidence Framework for Obesity Prevention Decision Making Institute of Medicine. Bridging the Evidence Gap in Obesity Prevention:A Framework to Inform Decision Making. The National Academies Press; 2010. 25032376

[11] Cartwright N, Hardie J. Evidence-Based Policy. A Practical Guide to Doing It Better. Oxford: Oxford University Press; 2012.

[12] Pawson R, Tilley N. Realistic Evaluation. London: Sage; 1997.

[13] United Nations (UN). World Economic Situation and Prospects. New York: United Nations; 2015.

[14] World Bank. World Development Indicators. http://data.worldbank.org/. Accessed July 4, 2015. Published 2015.

[15] World Health Organization (WHO). Noncommunicable disease country profiles 2014. http://www.who.int/nmh/countries/en/. Accessed July 4, 2015. Published 2014.

[16] Howitt C, Hambleton IR, Rose AM (2015). Social distribution of diabetes, hypertension and related risk factors in Barbados: a cross-sectional study. BMJ Open.

[17] World Health Organization (WHO). About WHO: Meet Dr St John, Executive Board Chairman. http://www.who.int/about/who_reform/change_at_who/dr_st_john/en/#.VZhnSflViko. Accessed July 4, 2015. Published 2013.

[18] Bernier NF, Clavier C (2011). Public health policy research: making the case for a political science approach. Health Promot Int.

[19] Dye TR. Understanding Public Policy. Engelwood Cliffs, NJ: Prentice Hall; 1972.

[20] Milio N (2001). Glossary: healthy public policy. J Epidemiol Community Health.

[21] Buse K, Mays N, Walt G. Making Health Policy. 2nd ed. Maidenhead, Berkshire: Open University Press; 2012.

[22] Kingdon J. Agendas, Alternatives, and Public Policies. New York: Harper Collins College Publishers; 1995.

[23] Exworthy M (2008). Policy to tackle the social determinants of health: using conceptual models to understand the policy process. Health Policy Plan.

[24] Craig RL, Felix HC, Walker JF, Phillips MM (2010). Public Health Professionals as Policy Entrepreneurs: Arkansas’s Childhood Obesity Policy Experience. Am J Public Health.

[25] Cairney P (2009). The role of ideas in policy transfer: the case of UK smoking bans since devolution. J Eur Public Policy.

[26] Kusi-Ampofo O, Church J, Conteh C, Heinmiller BT (2015). TResistance and change: a multiple streams approach to understanding health policy making in Ghana. J Health Polit Policy Law.

[27] Khayatzadeh-Mahani A, Sedoghi Z, Mehrolhassani MH, Yazdi-Feyzabadi V (2015). How Health in All Policies are developed and implemented in a developing country? A case study of a HiAP initiative in Iran. Health Promot Int.

[28] Jat TR, Deo PR, Goicolea I, Hurtig AK, San Sebastian M (2013). The emergence of maternal health as a political priority in Madhya Pradesh, India: a qualitative study. BMC Pregnancy Childbirth.

[29] Ridde V (2009). Ridde VPolicy implementation in an African State: an extension of Kingdon’s multiple-streams approach. Public Adm.

[30] Zahariadis N. The Multiple Streams Framework: structure, limitations, prospects. In: Sabatier P, ed. Theories of the Policy Process. 2nd ed. Cambridge MA: Westview Press; 2007:65-92.

[31] Start D, Hovland I. Tools for Policy Impact: A Handbook for Researchers. London: Overseas Development Institute; 2004.

[32] Caribbean Community Secretariat. Nassau Declaration on Health 2001 : The Health of the Region is the Wealth of the Region. http://www.caricom.org/jsp/communications/meetings_statements/nassau_declaration_on_health.jsp?menu=communications. Accessed July 7, 2015. Published 2001.

[33] Rose A, Hambleton I, Jeyaseelan S (2016). Establishing a national non-communicable disease surveillance system in a small-island state: Lessons learned from Barbados. Pan American J Public Health.

[34] Walt G, Shiffman J, Schneider H, Murray SF, Brugha R, Gilson L (2008). ‘Doing’ health policy analysis: methodological and conceptual reflections and challenges. Health Policy Plan.

[35] Milton K, Grix J (2015). Public health policy and walking in England—analysis of the 2008 ‘policy window’. BMC Public Health.

[36] Mc Hugh SM, Perry IJ, Bradley C, Brugha R (2014). Developing recommendations to improve the quality of diabetes care in Ireland: a policy analysis. Health Res Policy Syst.

[37] Takian A, Rashidian A, Kabir MJ (2011). Expediency and coincidence in re-engineering a health system: an interpretive approach to formation of family medicine in Iran. Health Policy Plan.

[38] El-Jardali F, Bou-Karroum L, Ataya N, El-Ghali HA, Hammoud R (2014). retrospective health policy analysis of the development and implementation of the voluntary health insurance system in Lebanon: learning from failure. Soc Sci Med.

[39] Cairney P, Jones MD (2016). Kingdon’s Multiple Streams Approach: What Is the Empirical Impact of this Universal Theory?. Policy Stud J.

[40] Butland B, Jebb S, Kopelman P, et al. Foresight. Tackling Obesities: Future Choices. Project Report. London: Goverment Office for Science; 2007. 10.1111/j.1467-789X.2007.00344.x 17316292

[41] Diepeveen S, Ling T, Suhrcke M, Roland M, Marteau TM (2013). Public acceptability of government intervention to change health-related behaviours: a systematic review and narrative synthesis. BMC Public Health.

[42] Huang TT, Cawley JH, Ashe M (2015). Mobilisation of public support for policy actions to prevent obesity. Lancet.

[43] Diem G, Brownson RC, Grabauskas V, Shatchkute A, Stachenko S (2015). Prevention and control of noncommunicable diseases through evidence-based public health: implementing the NCD 2020 action plan. Glob Health Promot.

[44] Alleyne G. Sectoral cooperation in the prevention and control of Noncommunicable Diseases. http://www.searo.who.int/entity/noncommunicable_diseases/events/ncd-bengaluru-sectoral-cooperation.pdf. Accessed July 17, 2015. Published 2014.

[45] McQueen DV, Wismar M, Lin V, Jones CM, Davies M, eds. Intersectoral Governance for Health in All Policies: Structures, actions and experiences. Copenhagen: World Health Organization; 2012. Observatory Studies Series.

[46] Sabatier P, Weible C. The advocacy coalition framework: innovations and clarifications. In: Sabatier P, ed. Theories of the Policy Process. 2nd ed. Cambridge MA: Westview Press; 2007:189-220.

[47] Edward G. Sinckler Tax Mix. Daily Nation. June 16, 2015.

